# Paper spray screening and liquid chromatography/mass spectrometry confirmation for medication adherence testing: A two‐step process

**DOI:** 10.1002/rcm.8553

**Published:** 2019-10-31

**Authors:** Catia Costa, Cecile Frampas, Katherine A. Longman, Vladimir Palitsin, Mahado Ismail, Patrick Sears, Ramin Nilforooshan, Melanie J. Bailey

**Affiliations:** ^1^ Surrey Ion Beam Centre University of Surrey Guildford GU2 7XH UK; ^2^ Department of Chemistry University of Surrey Guildford GU2 7XH UK; ^3^ Surrey and Borders Partnership Foundation NHS Trust, ACU Holloway Hill Chertsey KT16 0AE UK

## Abstract

**Rationale:**

Paper spray offers a rapid screening test without the need for sample preparation. The incomplete extraction of paper spray allows for further testing using more robust, selective and sensitive techniques such as liquid chromatography/mass spectrometry (LC/MS). Here we develop a two‐step process of paper spray followed by LC/MS to (1) rapidly screen a large number of samples and (2) confirm any disputed results. This demonstrates the applicability for testing medication adherence from a fingerprint.

**Methods:**

Following paper spray analysis, drugs of abuse samples were analysed using LC/MS. All analyses were completed using a Q Exactive™ Plus Orbitrap™ mass spectrometer. This two‐step procedure was applied to fingerprints collected from patients on a maintained dose of the antipsychotic drug quetiapine.

**Results:**

The extraction efficiency of paper spray for two drugs of abuse and metabolites was found to be between 15 and 35% (analyte dependent). For short acquisition times, the extraction efficiency was found to vary between replicates by less than 30%, enabling subsequent analysis by LC/MS. This two‐step process was then applied to fingerprints collected from two patients taking the antipsychotic drug quetiapine, which demonstrates how a negative screening result from paper spray can be resolved using LC/MS.

**Conclusions:**

We have shown for the first time the sequential analysis of the same sample using paper spray and LC/MS, as well as the detection of an antipsychotic drug from a fingerprint. We propose that this workflow may also be applied to any type of sample compatible with paper spray, and will be especially convenient where only one sample is available for analysis.

## 1 INTRODUCTION

Paper spray mass spectrometry was first described in the literature in 2010^1^ as a simple method for rapid analysis using a pre‐cut piece of paper to simultaneously extract and ionise a sample deposited on a porous substrate.^2^ The paper spray technique relies on the application of a voltage to a triangular piece of paper (with areas averaging 0.5 cm^2^), pre‐loaded with a sample and an extraction solvent. The voltage creates an electric field at the tip of the paper, resulting in the formation of charged droplets through a Taylor cone. The formed droplets, containing ions of the analyte, travel through the air, undergoing desolvation before entering a mass spectrometer.^1^, ^2^, ^3^, ^4^ Paper spray has been used qualitatively and quantitatively in many application areas from biological matrix analysis,^4^, ^5^, ^6^, ^7^, ^8^, ^9^, ^10^, ^11^, ^12^, ^13^, ^14^, ^15^ to foodstuff analysis,^16^, ^17^, ^18^, ^19^, ^20^, ^21^, ^22^, ^23^ explosives^24^, ^25^, ^26^, ^27^ and warfare agents.^28^, ^29^ Most recently, our group has reported the application of paper spray for the rapid detection of drugs of abuse in fingerprints^30^, ^31^ and explosives on swabs.^24^, ^25^

Despite the growing number of applications relating to paper spray, little consideration has been given to the extraction efficiency of the technique. Some studies have, however, explored the possibility of repeat analysis using paper spray. Bartella et al^19^ reported the detection of caffeine‐related substances in cocoa products, where the spray solvent was added in 15‐μL increments every 30 s for a 2 min acquisition. Zhang et al^32^ used paper spray for the quantitative analysis of therapeutic drugs in dried blood spots, showing that 40–70 extraction events were necessary for complete extraction of the analyte. Espy et al^34^ investigated the possibility of re‐analysing the same whole blood sample and found that re‐analysis did not affect the accuracy of the results, unless the concentration was close to the limit of quantitation. Conversely Shi et al^11^ explored the re‐analysis of whole blood samples for the detection of tacrolimus (immunosuppressive drug) and found that 25% of the re‐analysed samples (*n* = 40) produced spurious results. Vega et al^33^ and Bills et al^34^ also studied the effect of sample matrix, spray solvent, sample position and analysis substrate on the ion suppression effects and signal intensity of paper spray. Although the different parameters studied were shown to cause significant variation in ion suppression and signal intensity, the amount of extracted material was not considered. These reports highlighted the possibility of a second analysis from the same substrate and various factors affecting peak intensity, but none experimentally determined the extraction efficiency using an independent method.

One growing application area for mass spectrometry is treatment adherence monitoring. Reviews of non‐adherence^35^, ^36^, ^37^ reported that 50% of medicines are not being administered as prescribed by medical professionals. Mental health related disorders are one of the key areas where treatment adherence is problematic. It is estimated that one third of schizophrenic patients do not adhere to their prescribed treatment, which can ultimately lead to relapse and rehospitalisation. This has an economic impact not only on health services (A&E admission, inpatient costs), but also on the individual's well‐being and quality of life.^38^, ^39^, ^40^, ^41^

Fingerprints have been recently shown to be a useful sampling matrix for drug testing applications^30^, ^31^, ^42^, ^43^, ^44^, ^45^, ^46^, ^47^, ^48^, ^49^ due to the ease, safety, non‐invasiveness and rapidity of the sample collection. We have shown that illicit drugs and their metabolites can be detected in fingerprints rapidly using paper spray mass spectrometry, or via liquid chromatography/mass spectrometry (LC/MS). LC/MS carries the disadvantage of slower throughput than paper spray. On the other hand, the absence of a chromatography step in the paper spray process leaves it vulnerable to matrix effects as well as isobaric interferences, which may limit the sensitivity and selectivity of the technique compared with LC/MS. In the context of drug adherence monitoring, a false negative result has an impact for the relationship between patient and clinician, because it implies that a patient has not taken their medication. Therefore, any negative screening results should be confirmed.

In this paper, we explore the efficiency and repeatability of sample extraction of paper spray via subsequent extraction and LC/MS analysis using drugs of abuse as a model system. We then apply this two‐step process to show that in antipsychotic (quetiapine) adherence monitoring thef fingerprint can be first rapidly screened by paper spray mass spectrometry and negative screens can be explored further using LC/MS.

## 2 EXPERIMENTAL

### 2.1 Materials

Drug standards (cocaine, benzoylecgonine (BZE), heroin, 6‐acetylmorphine (6‐AM), cocaine‐d_3_, benzoylecgonine‐d_3_ (BZE‐d_3_), heroin‐d_9_ and 6‐acetylmorphine‐d_3_ (6‐AM‐d_3_)) were prepared from certified reference materials (Cerilliant, Round Rock, TX, USA) and were used to explore the extraction efficiency of paper spray mass spectrometry.

Certified reference materials of quetiapine, norquetiapine and quetiapine‐d_8_ were obtained from Sigma Aldrich(St Louis, MO, USA) and were used to develop paper spray and LC/MS methods for the analysis of fingerprint samples.

Optima™ LC/MS grade methanol (MeOH), dichloromethane (DCM), acetonitrile (ACN), isopropanol (IPA) and water (H_2_O) were used to prepare all solutions and solvent mixtures (Fisher Scientific, Madison, WI, USA). Formic acid or ammonium acetate (where applicable) was added to the mobile phase and spray solvents at 0.1% (v/v) or 10 mM (Fisher Scientific), respectively.

### 2.2 Experiments to measure paper spray extraction efficiency

#### 2.2.1 Paper spray analysis

Paper spray mass spectrometry was carried out using a custom‐made paper spray source as per our previous work.^25^, ^30^ The paper spray source was coupled to a Thermo Scientific™ Q Exactive™ Plus Hybrid Quadrupole‐Orbitrap™ mass spectrometer (Thermo Fisher Scientific, Hemel Hempstead, UK). To accommodate fingerprint samples, the analysis substrate (Whatman Grade 1 chromatography paper) was cut into a triangle shape (1.6 × 2.1 cm, base × height).

Positive ion mass spectra were acquired in full scan mode within a range of *m/z* 50–500 at a mass resolution of 280,000 at *m/z* 200 (unless otherwise stated), using 4 kV spray voltage, inlet temperature 250°C and S‐lens RF level 50. Standards of cocaine, BZE, heroin and 6‐AM were prepared at 250 ng/mL in ACN, spotted (5 μL) onto the analysis substrate and allowed to dry before being analysed by paper spray for 30 s, 60 s and 120 s, respectively.

#### 2.2.2 Post‐paper spray sample extraction

Following paper spray analysis, the paper triangles were placed in a 2‐mL Eppendorf microcentrifuge tube, with 1.5 mL of 10% DCM in MeOH. The tube was then centrifuged for 2 min (at 9.5 centrifugal force). The solvent extract was evaporated to dryness under a stream of nitrogen at room temperature (20°C) and reconstituted in 100 μL of a solution containing 95:5 water/acetonitrile + 0.1% formic acid + 7.5 ng/mL of internal standard, before being vortexed and transferred to a 300‐μL glass micro‐insert vial, with 5 μL injected onto an LC/MS system.

#### 2.2.3 Liquid chromatography/mass spectrometry

Chromatographic separation was performed on a Thermo Scientific™ Ultimate3000 UHPLC system, employing a Kinetex XB‐C18 column (100 × 2.1 mm, 5 μm) operated at 30°C at a flow rate of 0.25 mL/min. Gradient elution was performed with an initial mobile phase of 95:5 H_2_O/ACN (0.1% formic acid), increasing to 80:20 ACN/H_2_O (0.1% formic acid) over 2 min, and held constant for 0.5 min before returning to the initial mobile phase composition. The samples were introduced into the mass spectrometer using the standard electrospray ionisation (ESI) interface with an inlet temperature of 320°C and a spray voltage of 3 kV. Positive ion mass spectra were acquired in full scan mode within a range of *m/z* 50–500 at a mass resolution of 70 000 at *m/z* 200. Validation of the extraction and analysis procedure is described elsewhere.^42^ Monoisotopic masses of analytes were calculated using the Thermo Scientific monoisotopic mass calculator and these are documented in Table S1 (supporting information).

Calibration curves were determined by spiking paper substrates (of the same dimensions as those used for paper spray analysis) with 5 μL of analyte solutions in the 0–15 ng/mL range and extracting using the method described above. Figure S1 (supporting information) shows the resulting calibration curves for samples extracted and measured using LC/MS. The recovery efficiency of this LC method (shown in Table S2, supporting information) was calculated against two quality control solutions, directly deposited in LC/MS vials, of concentrations 7.5 and 12.5 ng/mL.

### 2.3 Application to fingerprint samples

#### 2.3.1 Fingerprint collection

A favourable ethical opinion for this project was obtained from the National Research Ethics Service (NRES‐REC reference: 18/NE/0071). Fingerprint samples were collected from patients at Surrey and Borders Partnership NHS Trust.

Fingerprint samples were collected on triangular pieces of paper (as described above) from the index, middle and ring fingers of the right hand. The fingertip was pressed onto the substrate for 10 s at a pressure of 1 kg (measured using a balance). The collected fingerprint samples were transported in a microscope glass storage case where they were stored at ambient temperature before analysis.

#### 2.3.2 Fingerprint analysis

For the sequential analysis of fingerprint samples using paper spray and LC/MS, the same instrumentation as described above was used. The paper spray and LC/MS methods were both adapted to increase the sensitivity to antipsychotics.

For paper spray, the optimised method used a spray solvent of 50 μL of 90:10 IPA/H_2_O with 0.1% (v/v) formic acid, 3.5 kV spray voltage and an inlet temperature of 320°C. Internal standard (quetiapine‐d_8_) was added prior to analysis and allowed to dry for 2 min. Spectra were acquired in full scan mode, in the range *m/z* 50–500, with a mass resolution of 140,000 at *m/z* 200. Data acquisition was set to 30 s in full scan mode, followed by 12 s in parallel reaction monitoring (PRM; resolution of 17,500 at *m/z* 200) mode for peak assignment confirmation. Validation for this paper spray method is reported in Figure S2, and Tables S3 and S4 (supporting information). The calibration equation fitted with R^2^ = 0.9839 and 0.9850 for quetiapine and norquetiapine, respectively, with the lowest detected mass at 50 pg and the relative standard deviation (RSD) between 6 and 34%, for both substances. No carryover or matrix effects were observed.

Following paper spray analysis, residual analytes were extracted from the paper using the same extraction method as above, but with a 100% MeOH extraction solvent and a reconstitution solvent of 50:50 ammonium acetate (pH 4.6)/ACN + 0.1% formic acid.

Confirmatory LC/MS analysis was also carried out under the same conditions as described above, but with an alternative gradient elution (initial mobile phase of 95:5 ammonium acetate (10 mM, pH 4.6)/ACN (0.1% formic acid), increasing to 10:90 ammonium acetate (10 mM, pH 4.6)/ACN (0.1% formic acid) over 2 min, held constant for 0.5 min before returning to the initial mobile phase composition). Data was acquired in positive ion mode, using full scan settings in the range *m/z* 120–500, with 70,000 mass resolution at *m/z* 200. An inlet temperature of 320°C and a spray voltage of 4 kV were employed.

Method validation for LC/MS is reported in Figure S3, Table S5, and Figures S4 and S5 (supporting information). The linearity (R^2^) of a calibration curve of analytes extracted from paper was 0.9932 and 0.9945 for quetiapine and norquetiapine, respectively, with a lowest mass detected of 10 pg and RSD between 7 and 22%, for both quetiapine and norquetiapine. No carryover or matrix effects were observed.

## 3 RESULTS AND DISCUSSION

### 3.1 Experiments to measure paper spray extraction efficiency

**FIGURE 1:**
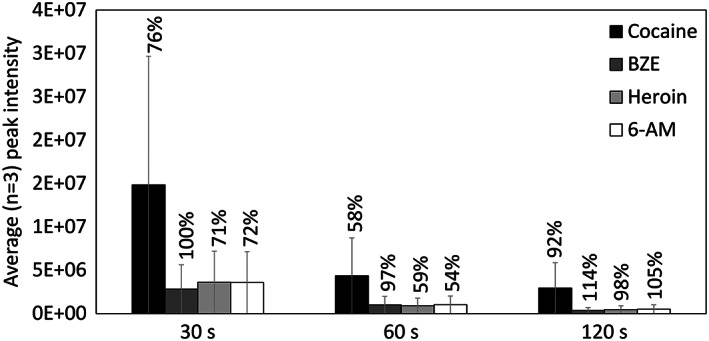
Average (*n* = 3) peak intensity of cocaine, BZE, heroin and 6‐AM measured in standards using paper spray mass spectrometry at different acquisition times of 30, 60 and 120 s. Percentages represent the relative standard deviation of the three repeat measurements

**FIGURE 2:**
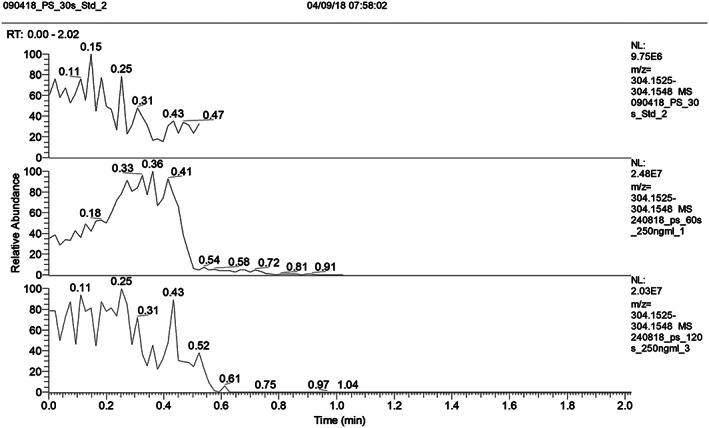
Example extracted ion chromatograms (XICs) for cocaine (*m/z* 304.1543) over acquisition times of 30 s (top), 60 s (middle) and 120 s (bottom)

Figure 1 shows the average (*n* = 3) peak intensity measured for cocaine, BZE, heroin and 6‐AM at different acquisition times. In each case, the analyte signals are highly variable, regardless of the acquisition time. The data shows that a longer acquisition time does not produce a higher average peak intensity. These results are corroborated by Figure 2, which shows example extracted ion chromatograms (XICs) for each time period. Under the conditions employed for these measurements, the analyte signal dropped at approximately 30 s. Therefore, either all the analyte is extracted from the paper in the first 30 s of analysis, or more spray solvent is needed to continue the extraction process. Alternatively, it has been observed by others that the spray will emerge from any sharp edge of the paper, not only the edge that is directly opposite the mass spectrometer inlet.^50^ It was therefore relevant to measure the extraction after different acquisition times in order to decouple these possibilities.

**FIGURE 3:**
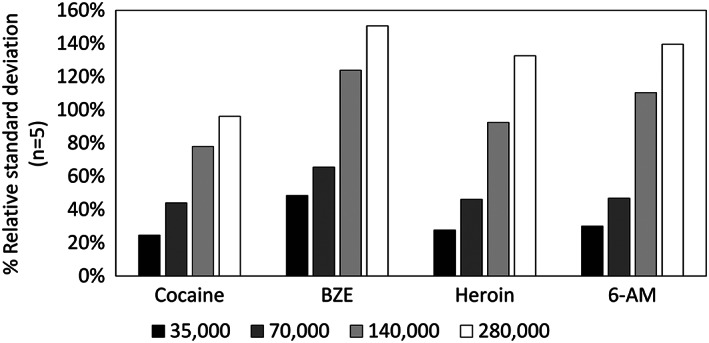
RSD% of the measured peak intensities of five replicate measurements at four different mass resolutions (35,000, 70,000, 140,000 and 280,000 at *m/z* 200) taken with paper spray

Figure 1 shows considerable measurement variability, and therefore the effect of mass resolution on the analyte signal was explored. Figure 3 shows the RSD of the measured peak intensities for five replicate measurements taken with paper spray at four different mass resolution settings over a 30 s acquisition. It is clear from Figure 3 that the mass resolution setting has a considerable effect on the measurement precision. This is explained by the longer transient times on the Orbitrap for measurements performed at 280,000 resolution (1024 ms), which results in a much lower number of scans for a given acquisition time than at 70,000 resolution (256 ms). Although lower resolution results in more precise measurements, it leaves the data open to isobaric interferences that may not be resolved. Whilst peak assignment can be confirmed by tandem mass spectrometry (MS/MS) measurements, this data shows that for paper spray Orbitrap mass spectrometry, there is a trade‐off between precision (due to fewer Orbitrap scans) and mass resolution.

**FIGURE 4:**
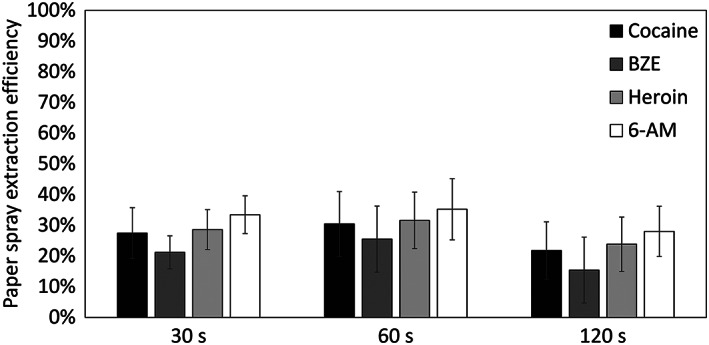
Percentage extraction efficiency of paper spray at 30, 60 and 120 s acquisition times. Error bars represent the standard deviation of three replicate extractions, each injected five times (*n* = 15)

Figure 4 shows that, regardless of the acquisition time, the extraction efficiency of analytes by paper spray was between 15 and 35%. This data shows that paper spray is a relatively inefficient process of analyte extraction, and corroborates what was shown in Figure 1, showing that a longer acquisition time does not result in more efficient extraction. Therefore, using this method, a 30 s acquisition leaves sufficient material for subsequent re‐analysis by paper spray or confirmation by LC/MS.

The paper spray and LC/MS methods used here have limits of detection (LODs) for cocaine of 20 pg and 10 pg, respectively.^31^, ^42^ This not only highlights the inherent higher sensitivity of the LC/MS method, but it shows an opportunity to use the low extraction efficiency of paper spray to advantage. If the mass of the analyte is near or just below the LOD of paper spray, subsequent analysis of the paper by LC/MS may allow detection of the substance after an initial paper spray screen.

### 3.2 Application to fingerprint samples

Fingerprints collected from two patients were sequentially analysed by paper spray and LC/MS. Patients 1 and 2 were on a maintained dose of 600 and 300 mg/day quetiapine, respectively.

**FIGURE 5:**
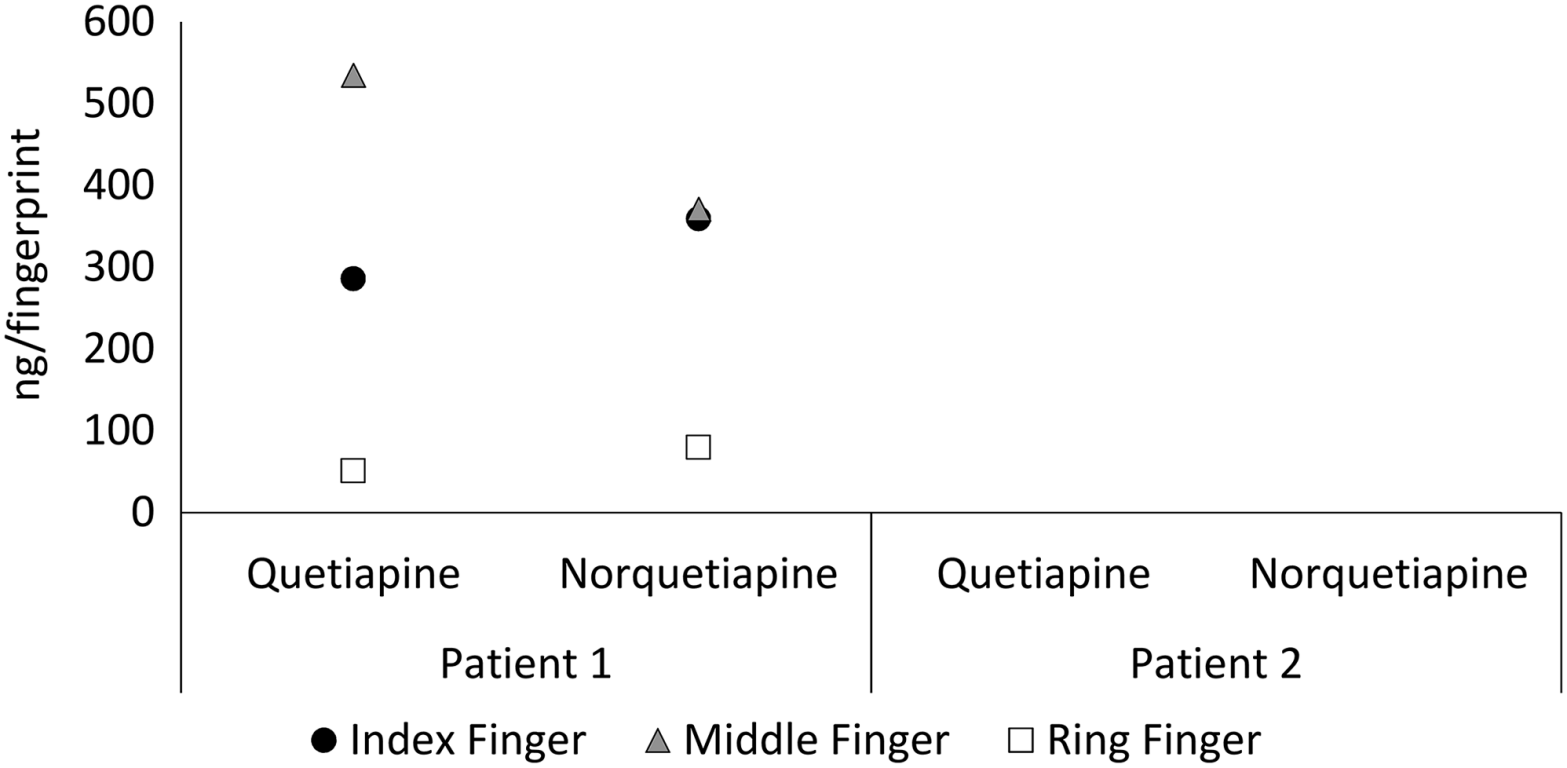
Paper spray results for the analysis of fingerprint samples collected from Patients 1 and 2 on a maintained dose of quetiapine (600 mg/day and 300 mg/day, respectively)

With paper spray, both quetiapine and its metabolite (norquetiapine) were detected in all fingerprints collected from Patient 1, as shown in Figure 5. No quetiapine or norquetiapine was detected in the samples of Patient 2, who was taking the lower dose of drug.

**FIGURE 6:**
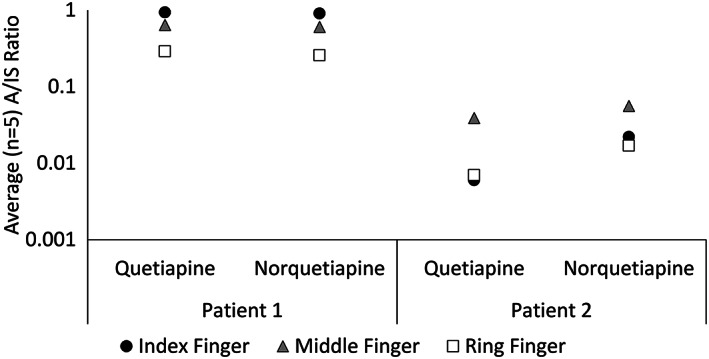
Average analyte‐to‐internal standard (a/IS) ratio (*n* = 5 injections) for the analysis of fingerprint samples collected from Patients 1 and 2 on a maintained dose of quetiapine (600 mg/day and 300 mg/day, respectively) after initial paper spray analysis

In contrast to the data from paper spray, sequential LC/MS analysis detected both substances in all three fingerprints in both patients, as shown in Figure 6. This demonstrates the enhanced sensitivity of LC/MS over paper spray, even though a proportion of the sample had been removed by previous analysis. In addition the analyte‐to‐internal standard (A/IS) ratio detected in Patient 2 is significantly lower than for Patient 1, consistent with the dose that was administered. Note that because the internal standard was added in the prior paper spray analysis, the data in Figure 6 cannot be reported in ng/fingerprint. Figures S4 and S5 (supporting information) show the overlay of the XICs for quetiapine and norquetiapine in blanks, standards and the fingerprint (right index) of Patient 2.

In the context of therapeutic drug monitoring (e.g. quetiapine and other antipsychotics), a high‐throughput (30 s) method like paper spray may be used as a fast screening tool for large volumes of samples. Samples returning a negative result using paper spray mass spectrometry can therefore be resolved using LC/MS. A limitation of the current work is the small number of patient samples that have been analysed. Future work will explore a greater number of patient samples to evaluate the usefulness of the approach for clinical applications.

This work is the first example of paper spray followed by LC/MS analysis and it has a wide range of potential applications, which could be explored in future work. For example, our recent work has shown the applicability of applying paper spray to materials collected on swabs for forensic applications.^25^ In this case, paper spray offers sufficient sensitivity, but subsequent LC/MS analysis would provide a characteristic retention time, allowing greater confidence in peak assignment.

## 4 CONCLUSIONS

We have demonstrated here that a sample previously analysed by paper spray mass spectrometry can be subsequently extracted and analysed by LC/MS. We have applied this process to the analysis of patient fingerprints, demonstrating the detection of antipsychotics in fingerprints for the first time. These findings have a wide range of possible applications, and here we illustrate how a negative screen for non‐adherence with antipsychotics using the rapid, but less sensitive paper spray mass spectrometry process can be confirmed using LC/MS.

## Supporting information

Table S1. Calculated monoisotopic mass of the analytes studied. Masses were calculated using the Thermo Scientific™ mass calculator.
Table S2. Extraction efficiency of the LC–MS sample preparation method for the analysis of cocaine, benzoylecgonine, heroin and 6‐acetylmorphine. Extraction efficiency was calculated using two extracted quality control standards (QC 1 = 7.5 ng/ml and QC 2 = 12.5 ng/ml) measured against a solution of the same concentrations.
Table S3. Lowest detected mass and associated RSDs for quetiapine and norquetiapine measured using the paper spray method developed for the detection of antipsychotics.
Table S4. Statistical comparison of analyte‐to‐internal standard (A/IS) ratio for antipsychotics and metabolites with and without the presence of fingerprint matrix using paper spray. In each case, F_calc_ and t_calc_ were less than the critical values.
Table S5. Lowest detected mass and associated RSDs for quetiapine and norquetiapine measured using the LC–MS method developed for the detection of antipsychotics.
Figure S1. Extracted calibration curves for (A) cocaine, (B) benzoylecgonine, (C) heroin and (D) 6‐acetylmorphine analysed using LC–MS.
Figure S2. Calibration curves for (A) quetiapine and (B) norquetiapine obtained using the optimised paper spray method of analysis (50 μl of 90:10 (%*v*/v) IPA:H_2_O + 0.1% formic acid and 3.5 kV applied voltage). The red dot represents the lowest detected mass, but was not included in the regression line.
Figure S3. Calibration curves for extracted standards from paper for (A) quetiapine and (B) norquetiapine analysed using LC–MS. The red dot represents the lowest detected mass.
Figure S4. Example extracted ion chromatograms for quetiapine (m/z 384.1740, retention time ~2.33 min) from (A) a blank solution containing only quetiapine‐d_8_, (B) a standard solution containing 500 pg of quetiapine, (C) a fingerprint collected from a participant not taking quetiapine and (D) a fingerprint from Patient 2 (right index) on a maintained dose of quetiapine.
Figure S5. Example extracted ion chromatograms for norquetiapine (m/z 384.1216, retention time ~2.33 min) from (A) a blank solution containing only quetiapine‐d_8_, (B) a standard solution containing 500 pg of norquetiapine, (C) a fingerprint collected from a participant not taking quetiapine and (D) a fingerprint from Patient 2 (right index) on a maintained dose of quetiapine.

## References

[1] Liu J , Wang H , Manicke NE , Lin JM , Cooks RG , Ouyang Z . Development, characterization, and application of paper spray ionization. Anal Chem. 2010;82(6):2463‐2471. 10.1021/ac902854g 20158226

[2] Liu J , Manicke NE , Zhou X , Cooks GR , Ouyang Z . Paper spray. In: Domin M , Cody R , eds. Ambient Ionization Mass Spectrometry. 1st ed. Cambridge: Royal Society of Chemistry; 2014. https://pubs.rsc.org/en/content/ebook/978‐1‐84973‐926‐9 Accessed July 30, 2019.

[3] Wang H , Liu J , Cooks RG , Ouyang Z . Paper spray for direct analysis of complex mixtures using mass spectrometry. Angew Chem Int Ed. 2010;49(5):877‐880. 10.1002/anie.200906314 20049755

[4] Espy RD , Manicke NE , Ouyang Z , Cooks RG . Rapid analysis of whole blood by paper spray mass spectrometry for point‐of‐care therapeutic drug monitoring. Analyst. 2012;137(10):2344‐2349. 10.1039/c2an35082c 22479698

[5] Kennedy JH , Palaty J , Gill CG , Wiseman JM . Rapid analysis of fentanyls and other novel psychoactive substances in substance use disorder patient urine using paper spray mass spectrometry. Rapid Commun Mass Spectrom. 2018;32(15):1280‐1286. 10.1002/rcm.8164 29757475

[6] Chiang S , Zhang W , Ouyang Z . Paper spray ionization mass spectrometry: Recent advances and clinical applications. Expert Rev Proteomics. 2018;15(10):781‐789. 10.1080/14789450.2018.1525295 30223684 PMC6320440

[7] McKenna J , Jett R , Shanks K , Manicke NE . Toxicological drug screening using paper spray high‐resolution tandem mass spectrometry (HR‐MS/MS). J Anal Toxicol. 2018;42(5):300‐310. 10.1093/jat/bky001 29377996

[8] Yannell KE , Kesely KR , Chien HD , Kissinger CB , Cooks RG . Comparison of paper spray mass spectrometry analysis of dried blood spots from devices used for in‐field collection of clinical samples. Anal Bioanal Chem. 2017;409(1):121‐131. 10.1007/s00216-016-9954-5 27822645

[9] Jett R , Skaggs C , Manicke NE . Drug screening method development for paper spray coupled to a triple quadrupole mass spectrometer. Anal Methods. 2017;9(34):5037‐5043. 10.1039/C7AY01009E

[10] Huang H , Wu Q , Zeng L , et al. Heating paper spray mass spectrometry for enhanced detection of propranolol in dried blood samples. Anal Methods. 2017;9(29):4282‐4287. 10.1039/C7AY01169E

[11] Shi RZ , El Gierari el TM , Manicke NE , Faix JD . Rapid measurement of tacrolimus in whole blood by paper spray‐tandem mass spectrometry (PS‐MS/MS). Clin Chim Acta. 2015;441:99‐104. 10.1016/j.cca.2014.12.022 25540885

[12] Espy RD , Teunissen SF , Manicke NE , et al. Paper spray and extraction spray mass spectrometry for the direct and simultaneous quantification of eight drugs of abuse in whole blood. Anal Chem. 2014;86(15):7712‐7718. 10.1021/ac5016408 24970379

[13] Su Y , Wang H , Liu J , Wei P , Cooks RG , Ouyang Z . Quantitative paper spray mass spectrometry analysis of drugs of abuse. Analyst. 2013;138(16):4443‐4447. 10.1039/c3an00934c 23774310 PMC3732448

[14] Wang H , Manicke NE , Yang Q , et al. Direct analysis of biological tissue by paper spray mass spectrometry. Anal Chem. 2011;83(4):1197‐1201. 10.1021/ac103150a 21247069 PMC3039116

[15] Manicke NE , Abu‐Rabie P , Spooner N , Ouyang Z , Cooks RG . Quantitative analysis of therapeutic drugs in dried blood spot samples by paper spray mass spectrometry: An avenue to therapeutic drug monitoring. J Am Soc Mass Spectrom. 2011;22(9):1501‐1507. 10.1007/s13361-011-0177-x 21953253

[16] Su Y , Ma X , Ouyang Z . Rapid screening of multi‐class antimicrobial residues in food of animal origin by paper spray mass spectrometry. Int J Mass Spectrom. 2018;434:233‐239. 10.1016/j.ijms.2018.10.003

[17] Zhou W , Yang Z , Huang S , Fang Z , Chen B , Ma M . Rapid quantitative analysis of ginkgo flavonoids using paper spray mass spectrometry. J Pharm Biomed Anal. 2019;171:158‐163. 10.1016/j.jpba.2019.04.018 30999226

[18] Yu M , Wen R , Jiang L , et al. Rapid analysis of benzoic acid and vitamin C in beverages by paper spray mass spectrometry. Food Chem. 2018;268:411‐415. 10.1016/j.foodchem.2018.06.103 30064777

[19] Bartella L , Di Donna L , Napoli A , Sindona G , Mazzotti F . High‐throughput determination of vitamin E in extra virgin olive oil by paper spray tandem mass spectrometry. Anal Bioanal Chem. 2019;411(13):2885‐2890. 10.1007/s00216-019-01727-z 30899998

[20] Teodoro JAR , Pereira HV , Sena MM , Piccin E , Zacca JJ , Augusti R . Paper spray mass spectrometry and chemometric tools for a fast and reliable identification of counterfeit blended Scottish whiskies. Food Chem. 2017;237:1058‐1064. 10.1016/j.foodchem.2017.06.062 28763950

[21] Mazzotti F , Di Donna L , Taverna D , et al. Evaluation of dialdehydic anti‐inflammatory active principles in extra‐virgin olive oil by reactive paper spray mass spectrometry. Int J Mass Spectrom. 2013;352:87‐91. 10.1016/j.ijms.2013.07.012

[22] Garrett R , Rezende CM , Ifa DR . Coffee origin discrimination by paper spray mass spectrometry and direct coffee spray analysis. Anal Methods. 2013;5(21):5944‐5948. 10.1039/C3AY41247D

[23] Deng J , Yang Y . Chemical fingerprint analysis for quality assessment and control of Bansha herbal tea using paper spray mass spectrometry. Anal Chim Acta. 2013;785:82‐90. 10.1016/j.aca.2013.04.056 23764447

[24] Costa C , van Es E , Sears P , et al. Exploring a route to a selective and sensitive portable system for explosive detection ‐ swab spray ionisation coupled to high‐field assisted waveform ion mobility spectrometry (FAIMS). Forensic Sci Int. 2019. 10.1016/j.fsisyn.2019.07.009 PMC7219150 32411973

[25] Costa C , van Es E , Sears P , et al. Exploring rapid, sensitive and reliable detection of trace explosives using paper spray mass spectrometry (PS‐MS). Propellants Explos Pyrotech. 2019;44(8):1021‐1027. 10.1002/prep.201800320

[26] Tsai CW , Tipple CA , Yost RA . Integration of paper spray ionization high‐field asymmetric waveform ion mobility spectrometry for forensic applications. Rapid Commun Mass Spectrom. 2018;32(7):552‐560. 10.1002/rcm.8068 29380926

[27] Tsai C‐W , Tipple CA , Yost RA . Application of paper spray ionization for explosives analysis. Rapid Commun Mass Spectrom. 2017;31(19):1565‐1572. 10.1002/rcm.7932 28681982

[28] McKenna J , Dhummakupt ES , Connell T , et al. Detection of chemical warfare agent simulants and hydrolysis products in biological samples by paper spray mass spectrometry. Analyst. 2017;142(9):1442‐1451. 10.1039/C7AN00144D 28338135

[29] Dhummakupt ES , Carmany DO , Mach PM , et al. Metal–organic framework modified glass substrate for analysis of highly volatile chemical warfare agents by paper spray mass spectrometry. ACS Appl Mater Interfaces. 2018;10(9):8359‐8365. 10.1021/acsami.7b19232 29411963

[30] Costa C , Webb R , Palitsin V , et al. Rapid, secure drug testing using fingerprint development and paper spray mass spectrometry. Clin Chem. 2016;63(11):1745‐1752. 10.1373/clinchem.2017.275578 28939761

[31] Jang M , Costa C , Bunch J , et al. On the relevance of cocaine and benzoylecgonine detection in a fingerprint. Under Review 10.1038/s41598-020-58856-0 PMC7005170 32029797

[32] Zhang Z , Xu W , Manicke NE , Cooks RG , Ouyang Z . Silica coated paper substrate for paper‐spray analysis of therapeutic drugs in dried blood spots. Anal Chem. 2012;84(2):931‐938. 10.1021/ac202058w 22145627 PMC3264786

[33] Vega C , Spence C , Zhang C , Bills BJ , Manicke NE . Ionization suppression and recovery in direct biofluid analysis using paper spray mass spectrometry. J am Soc Mass Spectrom. 2016;27(4):726‐734. 10.1007/s13361-015-1322-8 26729455

[34] Bills BJ , Kinkade J , Ren G , Manicke NE . The impacts of paper properties on matrix effects during paper spray mass spectrometry analysis of prescription drugs, fentanyl and synthetic cannabinoids. Forensic Chem. 2018;11:15‐22. 10.1016/j.forc.2018.08.002

[35] Kvarnström K , Airaksinen M , Liira H . Barriers and facilitators to medication adherence: A qualitative study with general practitioners. BMJ Open. 2018;8(1):e015332. 10.1136/bmjopen-2016-015332 PMC5786122 29362241

[36] Barnett NL . Medication adherence: Where are we now? A UK perspective. Eur J Hosp Pharm. 2014;21(3):181‐184. 10.1136/ejhpharm-2013-000373

[37] Kleinsinger F . The unmet challenge of medication nonadherence. Perm J. 2018;22:18‐033. 10.7812/TPP/18-033 PMC6045499 30005722

[38] Haddad PM , Brain C , Scott J . Nonadherence with antipsychotic medication in schizophrenia: Challenges and management strategies. Patient Relat Outcome Meas. 2014;5:43‐62. 10.2147/PROM.S42735 25061342 PMC4085309

[39] Higashi K , Medic G , Littlewood KJ , Diez T , Granström O , de Hert M . Medication adherence in schizophrenia: Factors influencing adherence and consequences of nonadherence, a systematic literature review. Ther Adv Psychopharmacol. 2013;3(4):200‐218. 10.1177/2045125312474019 24167693 PMC3805432

[40] Garcia S , Martinez‐Cengotitabengoa M , Lopez‐Zurbano S , et al. Adherence to antipsychotic medication in bipolar disorder and schizophrenic patients: A systematic review. J Clin Psychopharmacol. 2016;36(4):355‐371. 10.1097/jcp.0000000000000523 27307187 PMC4932152

[41] El‐Mallakh P , Findlay J . Strategies to improve medication adherence in patients with schizophrenia: The role of support services. Neuropsychiatr Dis Treat. 2015;11:1077‐1090. 10.2147/ndt.s56107 25931823 PMC4404876

[42] Ismail M , Stevenson D , Costa C , Webb R , de Puit M , Bailey M . Noninvasive detection of cocaine and heroin use with single fingerprints: Determination of an environmental cutoff. Clin Chem. 2018;64(6):909‐917. 10.1373/clinchem.2017.281469 29567660

[43] Bailey M , Randall EC , Costa C , et al. Analysis of urine, Oral fluid and fingerprints by liquid extraction surface analysis coupled to high resolution MS and MS/MS ‐ opportunities for forensic and biomedical science. Anal Methods. 2016;2016(16):3373‐3382. 10.1039/C6AY00782A 27990179 PMC5156400

[44] Muramoto S , Forbes TP , van Asten AC , Gillen G . Test sample for the spatially resolved quantification of illicit drugs on fingerprints using imaging mass spectrometry. Anal Chem. 2015;87(10):5444‐5450. 10.1021/acs.analchem.5b01060 25915085

[45] Bailey MJ , Bradshaw R , Francese S , et al. Rapid detection of cocaine, benzoylecgonine and methylecgonine in fingerprints using surface mass spectrometry. Analyst. 2015;140(18):6254‐6259. 10.1039/c5an00112a 25977942

[46] Kuwayama K , Tsujikawa K , Miyaguchi H , Kanamori T , Iwata YT , Inoue H . Time‐course measurements of caffeine and its metabolites extracted from fingertips after coffee intake: A preliminary study for the detection of drugs from fingerprints. Anal Bioanal Chem. 2013;405(12):3945‐3952. 10.1007/s00216-012-6569-3 23187828

[47] Goucher E , Kicman A , Smith N , Jickells S . The detection and quantification of lorazepam and its 3‐O‐glucuronide in fingerprint deposits by LC‐MS/MS. J Sep Sci. 2009;32(13):2266‐2272. 10.1002/jssc.200900097 19569106

[48] Jacob S , Jickells S , Wolff K , Smith N . Drug testing by chemical analysis of fingerprint deposits from methadone‐maintained opioid dependent patients using UPLC‐MS/MS. Drug Metab Lett. 2008;2(4):245‐247. 19356100 10.2174/187231208786734094

[49] Leggett R , Lee‐Smith EE , Jickells SM , Russell DA . "Intelligent" fingerprinting: Simultaneous identification of drug metabolites and individuals by using antibody‐functionalized nanoparticles. Angew Chem Int Ed Engl. 2007;46(22):4100‐4103. 10.1002/anie.200700217 17469083

[50] Espy RD , Muliadi AR , Ouyang Z , Cooks RG . Spray mechanism in paper spray ionization. Int J Mass Spectrom. 2012;325‐327:167‐171. 10.1016/j.ijms.2012.06.017

