# Supplementary material for: Paper spray screening and liquid chromatography/mass spectrometry confirmation for medication adherence testing: A two‐step process
Source: Rapid Commun Mass Spectrom. 2019 Oct 31;35(Suppl 2):e8553. doi: 10.1002/rcm.8553 (PMC8047880; doi:10.1002/rcm.8553)
Supplement: Supplementary file 1 — Table S1. Calculated monoisotopic mass of the analytes studied. Masses were calculated using the Thermo Scientific™ mass calculator. Table S2. Extraction efficiency of the LC–MS sample preparation method for the analysis of cocaine, benzoylecgonine, heroin and 6‐acetylmorphine. Extraction efficiency was calculated using two extracted quality control standards (QC 1 = 7.5 ng/ml and QC 2 = 12.5 ng/ml) measured against a solution of the same concentrations. Table S3. Lowest detected mass and associated RSDs for quetiapine and norquetiapine measured using the paper spray method developed for the detection of antipsychotics. Table S4. Statistical comparison of analyte‐to‐internal standard (A/IS) ratio for antipsychotics and metabolites with and without the presence of fingerprint matrix using paper spray. In each case, Fcalc and tcalc were less than the critical values. Table S5. Lowest detected mass and associated RSDs for quetiapine and norquetiapine measured using the LC–MS method developed for the detection of antipsychotics. Figure S1. Extracted calibration curves for (A) cocaine, (B) benzoylecgonine, (C) heroin and (D) 6‐acetylmorphine analysed using LC–MS. Figure S2. Calibration curves for (A) quetiapine and (B) norquetiapine obtained using the optimised paper spray method of analysis (50 μl of 90:10 (%v/v) IPA:H2O + 0.1% formic acid and 3.5 kV applied voltage). The red dot represents the lowest detected mass, but was not included in the regression line. Figure S3. Calibration curves for extracted standards from paper for (A) quetiapine and (B) norquetiapine analysed using LC–MS. The red dot represents the lowest detected mass. Figure S4. Example extracted ion chromatograms for quetiapine (m/z 384.1740, retention time ~2.33 min) from (A) a blank solution containing only quetiapine‐d8, (B) a standard solution containing 500 pg of quetiapine, (C) a fingerprint collected from a participant not taking quetiapine and (D) a fingerprint from Patient 2 (right index) on a [file RCM-35-e8553-s001.docx]

**Paper spray screening and LC-MS confirmation for medication adherence testing: a two-step process**

^a*^Catia Costa, ^b*^Cecile Frampas, ^b^Katherine A. Longman, ^a^Vladimir Palitsin, ^b^Mahado Ismail, ^b^Patrick Sears, ^c^Ramin Nilforooshan and ^b^Melanie J. Bailey

(*Joint first authors)

^a^Surrey Ion Beam Centre, University of Surrey, Guildford, GU2 7XH, UK; ^b^Department of Chemistry, University of Surrey, Guildford, GU2 7XH, UK; ^c^ Surrey and Borders Partnership Foundation NHS Trust, ACU, Holloway Hill, Chertsey, KT16 0AE, UK.

**Supplemental Data Table 1.** Calculated monoisotopic mass of the analytes studied. Masses were calculated using the Thermo Scientific^TM^ mass calculator.

|  | **Monoisotopic Mass [M+H]^+^** |
| --- | --- |
| **Cocaine** | 304.1543 |
| **Cocaine-d_3_** | 307.1732 |
| **Benzoylecgonine** | 290.1387 |
| **Benzoylecgonine-d_3_** | 293.1575 |
| **Heroin** | 370.1649 |
| **Heroin-d_9_** | 379.2214 |
| **6-acetylmorphine** | 328.1543 |
| **6-acetylmorphine-d_3_** | 331.1732 |
| **Quetiapine** | 384.1740 |
| **Quetiapine-d_8_** | 392.2242 |
| **Norquetiapine** | 296.1216 |

**Supplemental Data Table 2.** Extraction efficiency of the LC-MS sample preparation method for the analysis of cocaine, benzoylecgonine, heroin and 6-acetylmorphine. Extraction efficiency was calculated using two extracted quality control standards (QC 1 = 7.5 ng/ml and QC 2 = 12.5 ng/ml) measured against a solution of the same concentrations.

|  | **Cocaine** | | **Benzoylecgonine** | | **Heroin** | | **6-Acetylmorphine** | |
| --- | --- | --- | --- | --- | --- | --- | --- | --- |
|  | **QC1** | **QC2** | **QC1** | **QC2** | **QC1** | **QC2** | **QC1** | **QC2** |
| **Average** | 1.02 | 1.66 | 1.00 | 1.64 | 1.13 | 1.85 | 1.03 | 1.70 |
| **Standard Deviation** | 0.01 | 0.02 | 0.02 | 0.01 | 0.01 | 0.02 | 0.03 | 0.03 |
| **RSD%** | 1% | 1% | 2% | 1% | 1% | 1% | 3% | 2% |
| **Extraction efficiency** | 93% | 99% | 91% | 96% | 90% | 96% | 88% | 89% |

**(A)**

**(B)**

**(D)**

**(C)**

**Supplemental Data Figure 1.** Extracted calibration curves for (A) cocaine, (B) benzoylecgonine, (C) heroin and (D) 6-acetylmorphine analysed using LC-MS.

**Supplemental Data Figure 2.** Calibration curves for (A) quetiapine and (B) norquetiapine obtained using the optimised paper spray method of analysis (50 μl of 90:10 (%v/v) IPA:H_2_O + 0.1% formic acid and 3.5 kV applied voltage). The red dot represents the lowest detected mass, but was not included in the regression line.

**Supplemental Data Table 3.** Lowest detected mass and associated RSDs for quetiapine and norquetiapine measured using the paper spray method developed for the detection of antipsychotics.

|  | **Lowest detected mass (pg)** | **RSD%** |
| --- | --- | --- |
| **Quetiapine** | 50 | 14-34% |
| **Norquetiapine** | 50 | 6-14% |

**Supplemental Data Table 4.** Statistical comparison of analyte-to-internal standard (A/IS) ratio for antipsychotics and metabolites with and without the presence of fingerprint matrix using paper spray. In each case, F_calc_ and t_calc_ were less than the critical values.

| **Target Analyte** | **Analyte-to-internal standard (A/IS) ratio** | | **Matrix Effect (%)** | **F_calc_**  F_crit_ = 9.605  (P = 0.05) | ***t*_calc_**  *t*_crit_ = 2.26  (P = 0.05) |
| --- | --- | --- | --- | --- | --- |
|  | **Quality control** *^a^* | **In the presence of a fingerprint** *^a^* |  |  |  |
| Quetiapine | 0.207 ± 0.094 | 0.240 ± 0.070 | 15 | 1.81 | 0.61 |
| Norquetiapine | 0.395 ± 0.071 | 0.481 ± 0.152 | 22 | 4.54 | 1.14 |
| *^a^* mean ± standard deviation (n = 5). | | | | | |

**Supplemental Data Figure 3.** Calibration curves for extracted standards from paper for (A) quetiapine and (B) norquetiapine analysed using LC-MS. The red dot represents the lowest detected mass.

**Supplemental Data Table 5.** Lowest detected mass and associated RSDs for quetiapine and norquetiapine measured using THE LC-MS method developed for the detection of antipsychotics.

|  | **Lowest detected mass (pg)** | **RSD%** |
| --- | --- | --- |
| **Quetiapine** | 10 | 8-22% |
| **Norquetiapine** | 10 | 7-13% |

Quetiapine

Quetiapine

(A) Blank

(B) Standard solution (500pg)

(C) Blank fingerprint

(D) Patient fingerprint

**Supplemental Data Figure 4.** Example extracted ion chromatograms for quetiapine (m/z 384.1740, retention time ~2.33 min) from (A) a blank solution containing only quetiapine-d_8_, (B) a standard solution containing 500 pg of quetiapine, (C) a fingerprint collected from a participant not taking quetiapine and (D) a fingerprint from Patient 2 (right index) on a maintained dose of quetiapine.

Norquetiapine

Norquetiapine

(A) Blank

(B) Standard solution (500pg)

(C) Blank fingerprint

(D) Patient fingerprint

**Supplemental Data Figure 5.** Example extracted ion chromatograms for norquetiapine (m/z 384.1216, retention time ~2.33 min) from (A) a blank solution containing only quetiapine-d_8_, (B) a standard solution containing 500 pg of norquetiapine, (C) a fingerprint collected from a participant not taking quetiapine and (D) a fingerprint from Patient 2 (right index) on a maintained dose of quetiapine.
